# Editorial: Assessments and measures in psychotherapy research: going beyond self-report data

**DOI:** 10.3389/fpsyt.2023.1276222

**Published:** 2023-08-29

**Authors:** Brian Schwartz, Jessica Uhl, Dana Atzil-Slonim

**Affiliations:** ^1^Department of Psychology, University of Trier, Trier, Germany; ^2^Department of Psychology, Bar-Ilan University, Ramat Gan, Israel

**Keywords:** data-informed decision-making, video analysis, natural language processing, physiology, observational measures, psychological tasks

## Introduction

Good clinical decision-making during case conceptualization, treatment selection, and the adjustment of the therapeutic strategy over the course of treatment can improve the effectiveness of psychotherapy. To support therapists making such decisions, measurement-based and data-informed psychological therapy, which relies on prediction algorithms, can be implemented (1). In recent decades, the statistical methods used to create these algorithms have improved rapidly, e.g., with the introduction of machine learning (ML) into psychotherapy research (2). However, these advanced methods quickly reach their limits when the data base is insufficient to realize their full potential to predict outcomes and derive clinical recommendations. Indeed, psychotherapeutic processes are rich on various channels, including verbal and nonverbal exchanges between patients and therapists, emotional expressions, somatic-motor activity, and physiological processes. These different modalities convey important information about patients' mental states, thoughts, and emotions and can lead to a more in-depth understanding of psychotherapy processes and outcomes. However, most measurement-based prediction algorithms have been calculated based on self-report data. Although they are the cornerstone of psychotherapy research, standardized subjective self-report measures have critical shortcomings, including limited patient self-insight, response tendencies, and cognitive biases (e.g., memory bias) (3, 4). To better support therapists with the most accurate models and recommendations, we must go beyond self-report questionnaires. In the following sections, we will summarize assessments and measures in psychotherapy research that can capture important information about patients and treatment in addition to and beyond self-report questionnaires, thus providing novel data for evidence-based and data-informed psychotherapy.

## Video and observer-based data

With advancing technology and digitization, video recordings can provide more objective information about the patient, therapist, and treatment non-invasively and with high temporal resolution. Observation methods can be applied to video recordings to capture verbal information and behavior (e.g., therapeutic interventions) (5), as well as non-verbal cues, such as movement (e.g., movement-based attunement) (6), gestures, and facial expressions. Methodological studies evaluating the validity of these measures are crucial to gain insight into what we are assessing (7). Three papers in the Research Topic examine video- and observer-based methods. Maaß et al. validated a brief rating scale to reliably and applicably assess basic psychotherapeutic communication skills in clinical training. Diaz et al. applied an observational coding system measuring relationship-building behaviors between therapist and clients and modeled affective dynamics within the dyads. Terhürne et al. introduce a video-analysis system for automated emotion recognition and examined associations between system ratings of emotional valence and arousal and self-reports, change processes, and symptoms.

## Audio and text data

Each video recording also provides one or more audio tracks containing verbal and paraverbal information. Linguistic expressions in a session reveal patient and therapist thoughts and emotions and provide information about the dyadic interaction, which can be extracted and analyzed via natural language processing. Speech content is transcribed either manually by human raters or automatically and thus transferred into text form. These texts can be evaluated by qualitative linguistic analyses or according to predefined categories such as sentiments (8). Paraverbal parameters, e.g., speech rate, speech frequency, and vocal arousal, can be extracted from the audio files to examine intra- and interpersonal emotion dynamics and other therapeutic processes (9, 10). Four papers in this Research Topic analyze verbal or paraverbal features. Broadbent et al. identified clients at risk of suicide using natural language processing on data from a text-based crisis encounter and mobile tipline app. Egozi et al. applied observational measures of attachment and therapeutic distance to transcripts of video-recorded patient and therapist narratives about their therapeutic relationship. Lee et al. examined transcribed session recordings regarding the use of discourse particles, which indicate the formality in language, and its association with observer-rated therapist empathy. Opladen et al. validated fundamental frequency *f* 0, which is a commonly used index for emotional activation, as a marker of arousal, valence, and distress during a body exposure session.

## Physiological data

Considering the close association between psychological and physical processes, biological and physiological variables can offer further useful information. Variables such as heart rate variability (HRV) or electrodermal activity (EDA) allow an objective and continuous recording of stress or emotional arousal during the therapy session. Additionally, patient and therapist co-activate and co-regulate their physiological responses (11). The availability of smartwatches and other wearable devices means that these measurements no longer represent a noticeable intrusion into the therapeutic setting (12). Three papers in this Research Topic rely mainly on data from physiological measures. Andorfer et al. assessed the psychophysiological stress response during a social-evaluative speaking task via HRV, heart rate, and blood pressure to evaluate the effectiveness of a mindfulness-based intervention. Nyman-Salonen et al. provide a narrative review of opportunities and challenges associated with measuring embodied variables in psychotherapy, which focuses on the sympathetic nervous system and body movements. Looking to the future, Hollandt et al. present the protocol of a pilot study planning to examine the dyadic synchrony of heart rate, EDA, and electroencephalogram via wearable devices during two experimental emotion-processing tasks.

## Experimental tasks

The measures discussed above can be collected passively before, during, and after treatment. Patients do not have to do anything other than participate in the regular course of therapy, meaning that these assessments often involve less effort for patients than self-report questionnaires. However, additional information about patients can also be obtained via more active assessment methods such as experimental tasks. Psychological tasks can, for example, include implicit association tests as an indirect measure of pathological attitudes and cognitions or change processes such as outcome expectations (13). In this Research Topic, one paper applied an experimental task. Amano et al. assessed reaction times and the ratio of the number of responses for positive valence in the future thinking task as potential objective measures of treatment process and outcome.

## Conclusion

This Research Topic highlights the range of possible assessments and measures in psychotherapy that go beyond self-reports. The 11 studies aimed at validating these measures as well as measuring and predicting treatment processes and outcomes. The next step must involve the multimodal and multimethod assessment of patient and treatment information. Some studies included here have already collected measures from varying channels (e.g., Opladen et al.), and introduce methods to integrate information from different modalities (e.g., Nyman-Salonen et al.). With the advancing digitization and technologization of psychotherapeutic settings, the implementation of these measures into routine care is becoming more feasible. Nevertheless, it requires time, resources, and improved scientific training for practitioners to be able and feel confident making clinical decisions supported by these new sources of data (1).

## Author contributions

BS: Conceptualization, Writing—original draft, Writing—review and editing. JU: Writing—review and editing. DA-S: Writing—review and editing.
